# Does the messenger make the difference? Perceived reliability of health information sources during the COVID-19 pandemic in multi-ethnic Malaysia

**DOI:** 10.1371/journal.pgph.0007397

**Published:** 2026-10-01

**Authors:** Nicholas Yee Liang Hing, Yew Kong Lee, Hyung Joon Kim, Nurhyikmah M. Lothfi, Elizabeth Wong, Joan Costa-Font

**Affiliations:** 1 Centre for Clinical Epidemiology, Institute for Clinical Research, National Institutes of Health, Ministry of Health, Shah Alam, Malaysia; 2 Department of Primary Care Medicine, Faculty of Medicine, University Malaya, Ministry of Higher Education, Petaling Jaya, Malaysia; 3 United Nations Children’s Fund, Country Office, Putrajaya, Malaysia; 4 Department of Health Policy, London School of Economics and Political Science, London, United Kingdom; Universiti Kuala Lumpur Royal College of Medicine Perak, MALAYSIA

## Abstract

The COVID-19 infodemic highlighted the need to identify the extent to which health information messengers were perceived as reliable by the public in supporting effective public health communication. A question that remains is whether there are differences across ethnic groups in information processing. This study examines the perceived reliability of three formal sources (healthcare workers, scientists, and non-governmental organisations [NGOs]) and four informal sources (religious leaders, community leaders, interpersonal sources, and influencers) among Malaysia’s multi-ethnic population through a cross-sectional online survey conducted between April and June 2021. Logistic regression was used to identify the perceived reliability of different information sources in steering vaccination intentions. We further examine the extent to which there were ethnic differences in the perceived reliability of these sources across education levels and age groups. Adjusted predicted probabilities were estimated to illustrate these differences. Among 5,784 participants, formal sources were consistently perceived as the most reliable across all ethnic groups and, together with community leaders, were identified as the main information sources influencing vaccination intention. We find large predicted probabilities of perceiving healthcare workers and scientists as reliable across most groups (≥ 80%), except among Indigenous groups who exhibit lower education attainment and are older (≤ 78%). Across education levels and age groups, Indians and Chinese exhibit higher predicted probabilities of perceiving NGOs as reliable, averaging approximately 80% and 75%, respectively, compared with approximately 65–70% among Malays and Indigenous groups. In contrast, predicted probabilities of perceiving community leaders as reliable were moderate across all ethnicities at the lowest education level and youngest age group (45 – 55%). This pattern remained relatively stable across higher education levels and age groups among Malays and Indigenous groups, but generally decreased among Chinese and Indian respondents. These results suggest that healthcare workers and scientists ought to remain central to health communication strategies, while NGOs and community leaders may play complementary roles. Multi-messenger approaches may enhance the effectiveness of public health responses during health crises.

## Introduction

Communicating health information at a critical time such as a pandemic can play a pivotal role in shaping population behaviour, especially in adopting preventive measures such as medical screening and vaccination, and seeking care for early detection of symptoms and appropriate medical treatment [1,2]. Access to accurate and timely health information is crucial for empowering individuals to make informed decisions about their health, which can significantly impact the overall well-being of a population. However, emergency situations are often characterised by time and resource constraints, thereby requiring public health practitioners to strategically communicate using the most effective information sources to maximise public reach, influence, and behavioural impact.

Source Credibility Theory posits that the persuasive impact of communication is largely determined by the perceived credibility of its source [3,4]. Credibility is commonly conceptualised as comprising key dimensions such as expertise and trustworthiness, whereby sources perceived as knowledgeable, competent, and honest are more likely to be persuasive and to influence individuals’ attitudes and decisions. Studies based on this theory have shown that information from sources perceived as credible is associated with a greater intention of adopting a recommended health behaviour [5–8]. Advice or information delivered by credible sources is more likely to be accepted and acted upon, thereby eliciting behavioural change through enhanced motivation and by shaping key cognitive appraisals such as perceived susceptibility and severity of health outcomes, in line with the Health Belief Model [9,10]. Beyond these dimensions, individuals often form an overall evaluative judgment of the reliability of information provided by a source. This evaluation directly reflects on the perceived reliability of an information source, as observed by the reliability of the information it communicates. It represents individuals’ overall assessment of the source’s credibility and the extent to which it is perceived as capable of providing trustworthy information worthy of acceptance [11].

The perceived reliability of health information sources is an important mediator between information exposure and health behaviours [12,13]. Extensive research has been conducted to assess the reliability of various sources of health information [14,15]. Information sources can be categorised broadly into two forms: the channel, which refers to the medium through which the information is delivered (e.g., social media, the internet, television, newspapers), and the messenger, which refers to the individuals or groups providing the information (e.g., doctors, pharmacists, friends, relatives). Messengers can be further categorised into two types: formal and informal sources [11]. Formal sources refer to structured or professionally oriented messengers that convey information grounded in expertise or organisational authority. In contrast, informal sources are socially oriented messengers who convey information through social influence or community networks rather than institutional or professional authority.

Health communication studies often assess communication channels and messengers together when examining perceived reliability [11,15–18]. This conflation limits the capacity to isolate and measure the perceived reliability of information from specific messengers, independent of the communication channels through which the information is delivered. Consequently, a more focused examination of messengers is warranted to better understand their unique contribution to perceived information reliability. However, identifying messengers perceived as providing reliable information alone may be insufficient to fuel effective health information campaigns that influence behaviour, as discrepancies between favourable perception and actual behaviour may arise under different contextual circumstances [19–21]. It is therefore equally important to determine how perceptions of information reliability from various messengers are associated with the behaviour of interest. Such insights would enable health communicators to strategically engage priority messengers as information sources to enhance the effectiveness of public health communication.

A key component of effective health communication campaigns is understanding how to tailor message dissemination strategies to engage the public in adopting recommended public health measures while countering the spread of misinformation and disinformation. This is especially pertinent during health emergencies amidst an ongoing infodemic [22]. Under such circumstances, health authorities need to design communication campaigns that employ priority messengers perceived as reliable sources of information across diverse segments of the population.

Malaysia is a multi-ethnic country where Malays constitute the majority population, followed by Chinese, Indians, and various Indigenous ethnic groups residing in both Peninsular and East Malaysia. The coexistence of diverse cultural backgrounds provides a valuable setting for exploring variation in perceived reliability of information sources across ethnic and sociodemographic groups. Previous studies have shown that age and educational attainment are important determinants of perceptions toward health information sources [17,23,24]. As these sociodemographic influences may operate differently across cultural and ethnic contexts, the associations between age, educational attainment, and perceived reliability of information sources may not be uniform across ethnic groups.

Based on these important considerations and the potential to draw valuable insights that can inform future health communication strategies, we conducted a study using the COVID-19 pandemic as a contextual backdrop to achieve several objectives. Specifically, this study aimed to examine how Malaysians, both as a whole and across the major ethnic groups, perceived the reliability of health information messengers from three formal sources—healthcare workers, scientists, and non-governmental organisations (NGOs)—and four informal sources—religious leaders, community leaders, interpersonal sources (friends, relatives and acquaintances), and influencers. We subsequently identified health information messengers whose perceived reliability as a COVID-19 information source was positively associated with COVID-19 vaccination intention and further examined ethnic differences in perceived reliability across education levels and age groups.

## Materials and methods

### Study design

We conducted a secondary cross-sectional analysis of participants from a previously conducted randomised controlled trial (RCT). The initial experiment investigated how different message framing approaches influenced participants’ intentions to receive and recommend the COVID-19 vaccine [25]. None of the intervention messages promoted or identified any particular information source or messenger as a reliable source of COVID-19 information. Perceived reliability of information sources was not an intervention target and was therefore not expected to differ systematically according to intervention allocation. Additionally, there were no statistically significant differences in perceived reliability of the examined information sources across study arms (Table A in S1 Appendix). Participants were therefore pooled across study arms for the present secondary analysis.

After completing the section related to the experiment, participants continued answering a survey that included examining the perceived reliability of COVID-19 information from various sources. It’s worth mentioning that given that the dataset was obtained from an RCT designed for a different primary outcome, the analyses reported here are exploratory and intended to be hypothesis-generating.

The experiment and survey were administered via Dynata, an American-based international market research company that hosts online surveys. Dynata’s platform includes a panel of Malaysian participants who voluntarily sign up to participate in surveys in exchange for reward points. The use of Dynata’s platform allowed for efficient recruitment and data collection from a large diverse sample of adult Malaysians. This web-based approach was particularly suitable given the ongoing COVID-19 pandemic and related social distancing measures, allowing participants to complete the survey safely in a remote environment.

### Study setting

The survey was conducted in Malaysia, an upper-middle-income country in Southeast Asia. In 2021, the country reported an estimated population of approximately 30 million Malaysian citizens, comprising about 56.8% Malays, 23.2% Chinese, 6.7% Indians, and 12.6% Indigenous ethnic groups, with the remaining 0.7% made up of other minor ethnic groups [26].

The present survey spanned over a six-week period, from April 29 to June 7, 2021. This time-frame coincided with the initial stages of the country’s national COVID-19 vaccination programme. The recruitment process targeted individuals aged 18 years and above, who had not received any dose of the COVID-19 vaccine at the time of the survey. Participants were required to be proficient in either English or Malay to ensure they could understand and respond to the survey questions.

### Survey instrument

The questionnaire used in the survey was mainly developed by adapting items from questionnaires used in previous studies conducted in Malaysia that examined COVID-19 vaccination preferences and vaccine acceptance [27,28]. The item assessing intention to receive the COVID-19 vaccine was adopted from a previously published international study [29]. The developed questionnaire was made available in both English and Malay. Where Malay versions of the relevant questionnaire sections were not available, the items were translated into Malay and back-translated into English to ensure consistency of meaning between the two language versions. The questionnaire subsequently underwent content and face validation by a multidisciplinary panel comprising two public health researchers, three health communication experts, and two health behaviour researchers. The questionnaire was then pilot tested among 10 members of the public to assess the clarity, comprehensibility, and appropriateness of the items and response options before data collection.

### Data collection

Data regarding baseline intention to accept the COVID-19 vaccine and general attitude towards vaccines were first collected from participants before they were subjected to a brief message intervention. Details regarding these measures can be found in the published experimental phase of the study [25]. Baseline intention was measured using a question that asked participants whether they would accept the COVID-19 vaccine, rated on a four-point Likert scale: “Definitely no”, “Not sure, but probably no”, “Not sure, but probably yes”, and “Definitely yes”. Using a five-point Likert scale, general attitude toward vaccines was assessed based on participants’ responses regarding the perceived efficacy of vaccines in preventing serious diseases, and whether vaccination is supported by their religious or cultural beliefs. Where applicable, participants were also asked whether they had ever refused vaccination for their child. Participants were considered to have a negative attitude if they expressed disagreement, uncertainty, or refusal in response to any of the items.

After the experimental phase, participants were asked “*In your opinion, how reliable is the information on COVID-19 from the following sources?*” and then rated their perceived reliability using a 5-point Likert scale ranging from “Strongly Not Reliable” to “Strongly Reliable”, from seven sources: healthcare workers, scientists, non-governmental organisations (NGOs), religious leaders, community leaders, interpersonal sources (friends, relatives, and acquaintances), and influencers. These ratings determined the perceived reliability of investigated health information sources.

In addition, participants were asked whether they trusted the government to manage the COVID-19 situation effectively by rating their agreement with the statement “*Overall, I trust the Government for the management of COVID-19*”. Participants rated their agreement using a 5-point Likert scale, ranging from “Strongly Disagree” to “Strongly Agree.” This question was included to capture participants’ broader trust in public institutions, as trust in government has been shown to influence perceptions of public health messages and the likelihood of believing in misinformation [30,31]. Sociodemographic characteristics (i.e., age, sex, ethnicity, educational level, and income level) were then collected after these surveys.

### Statistical analysis

Descriptive statistics were used to summarise the demographic and response data. For ease of analysis, age was categorised into age groups: 18–30, 31–40, 41–50, 51–60, and above 60. Education level was categorised as low (Secondary education and below), intermediate (Diploma, Certification or Pre-university courses) and high (tertiary level education). To facilitate interpretation of the regression estimates and distinguish participants who clearly perceived a source as reliable from those who did not, all Likert scale responses were dichotomised to allow for subsequent analyses using logistic regression. Negative or uncertain ratings were categorised under a negative category, while positive ratings were grouped under a positive category. For example, responses of “Strongly Not Reliable,” “Not Reliable,” and “Not Sure” in the reliability assessment were combined into a single category labelled “Not Reliable,” whereas responses of “Reliable” and “Strongly Reliable” were categorised as “Reliable”. The proportion of participants who perceived each information source as reliable was descriptively analysed overall and across ethnicities.

Multivariable logistic regression was conducted to identify information sources that were perceived as providing reliable COVID-19 information and were significantly associated with greater odds of intending to receive the COVID-19 vaccine. These sources were labelled as information sources positively associated with vaccination intention. Baseline COVID-19 vaccination intention was used as the dependent variable because it was assessed before participants were exposed to the intervention in the RCT, which was designed to influence vaccination intention. Baseline intention responses were dichotomised into two categories: ‘positive’ for those who answered “Definitely yes”, and “Not sure, but probably yes”, and ‘negative’ for all other responses which were considered negative (i.e., “Definitely no” and “Not sure, but probably no”). Each investigated source acted as binary predictors in the regression model, with the model adjusted for general attitude toward vaccines. To examine the robustness of the findings, the models were further adjusted for sex, age group, education level, income level, and trust in the government. Results were presented as odds ratios (OR) with robust standard errors to assess the strength and direction of associations between examined factors and the perceived reliability of different information sources, with significant associations marked by p-values less than 0.05.

We further explored how these identified information sources should be tailored to different ethnic communities in Malaysia across education levels and age groups. Logistic regression models were fitted to examine the interactions of ethnicity with education level, while using perceived reliability of each identified information source as the outcome variable. Models were adjusted for age group, sex, income level, trust in the government, and general attitude toward vaccines. Another set of interaction models was generated between ethnicity and age group, adjusting for the same set of variables, including education level. However, due to data scarcity among Indigenous groups, the 41–50, 51–60, and ≥60 age groups were merged into a single category (≥41) for these models. Adjusted predicted probabilities were then estimated from the fitted logistic regression models by averaging predictions over the observed distribution of the remaining covariates to estimate each ethnic group’s probability of perceiving each identified information source as reliable across the interacting sociodemographic characteristics. All analyses were conducted using Stata version 16.

### Ethics statement

This study has been registered with the Malaysian National Medical Research Register (NMRR-21-26-58107 (IIR)) and received ethical approval from the Medical Research and Ethics Committee of the Ministry of Health Malaysia (Ref: KKM/NIHSEC/ P21-130). The study was conducted in compliance with ethical principles outlined in the Declaration of Helsinki and Malaysian Good Clinical Practice Guideline. Prior to enrollment, all potential study participants were presented a page that described information about the study. Consenting participants provided informed consent by clicking on a button indicating agreement to join the study.

## Results

In total, 5,784 participants responded to the survey. Their sociodemographic characteristics are summarised in Table 1. The sociodemographic profile of the study sample was broadly comparable to those of the Malaysian population in terms of sex, household income, and ethnicity, although Indigenous ethnic groups were underrepresented by approximately half compared with national population estimates [32,33]. However, due to the survey’s online nature, the proportion of participants in the two youngest age groups (18–40) was approximately 70% higher than the national average [33]. Additionally, our sample was skewed towards individuals with higher education. The proportion of total participants with more than a secondary education level exceeds the national proportion by more than three times. Trust in the government’s efforts to manage the pandemic effectively was high, with approximately three-quarters of respondents expressing a positive response. A large majority of the participants had a positive intention to receive the COVID-19 vaccine.

**Table 1:** Sociodemographic characteristics of participants (n = 5,784).

Characteristics	n (%)
Age group	
18-30	2,306 (39.9%)
31-40	1,545 (26.7%)
41-50	1,286 (22.2%)
51-60	445 (7.7%)
≥ 61	202 (3.5%)
Sex	
Male	2,907 (50.3%)
Female	2,877 (49.7%)
Ethnicity	
Malay	3,399 (58.8%)
Chinese	1,579 (27.3%)
Indian	519 (9.0%)
Indigenous groups	287 (5.0%)
Household income level ^a^	
B40 (≤ RM 4,849)	2,526 (43.7%)
M40 (RM 4,850 – RM 10,959)	2,443 (42.2%)
T20 (≥ RM 10,960)	815 (14.1%)
Education level	
Low	1,192 (20.6%)
Intermediate	1,727 (29.9%)
High	2,865 (49.5%)
Trust in government handling COVID-19 situation	
Yes	4,205 (72.7%)
No	1,579 (27.3%)
Intention to receive COVID-19 vaccination	
Definitely not	106 (1.83%)
Not sure, but probably not	390 (6.74%)
Not sure, but probably yes	1,724 (29.81%)
Definitely yes	3,564 (61.62%)

a. USD equivalents were estimated using the average 2021 exchange rate (RM 1 = USD 0.24).

Fig 1 presents the proportion of participants who perceived each of the seven information sources as either reliable or not reliable. The full distribution of responses across the 5-point Likert scale is presented in Table B in S1 Appendix.

**Fig 1:**
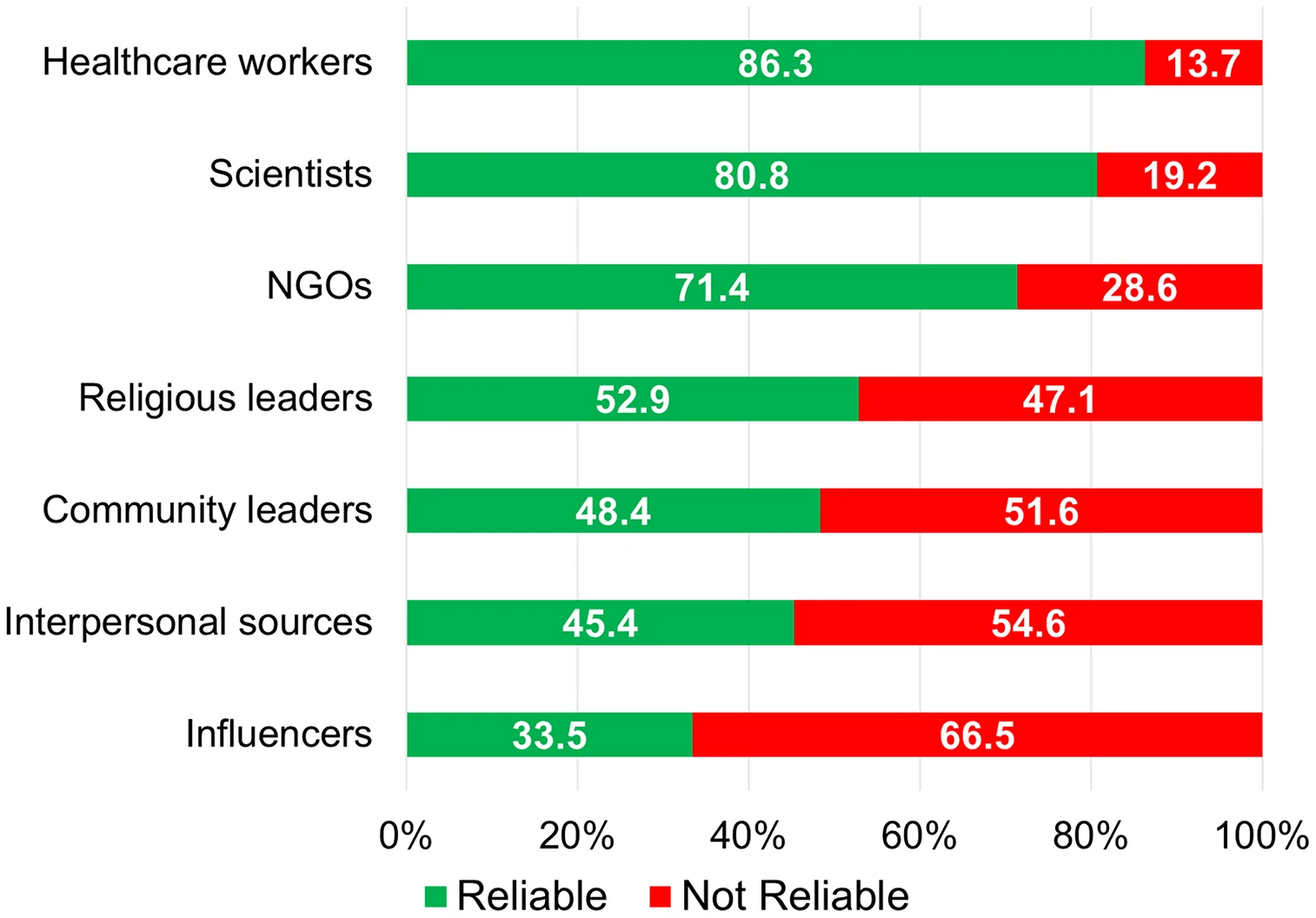
Proportion of participants responding towards perceived reliability of different information sources.

Healthcare workers are perceived to be the most reliable source for COVID-19 information, followed by Scientists, and NGOs. More than 70% of participants perceive these sources as reliable. There was a substantial fall in proportion of participants perceiving the other sources as reliable, with approximately 50% perceiving religious leaders, community leaders, and interpersonal sources as reliable, while slightly more than 30% of participants found influencers to be reliable.

Fig 2 presents the proportion of participants in each ethnic group who perceived each information source as reliable or not reliable. The corresponding full distribution of responses across the 5-point Likert scale is provided in Table C in S1 Appendix. Malays had the highest proportion of participants who perceived most sources as reliable compared to other ethnicities. A particularly high proportion of Malays (almost 70%) perceive religious leaders as reliable as compared to other ethnic groups (Chinese: 27%, Indians: 37%, Indigenous groups: 52%). The Chinese respondents reveal a more reserved perception of reliability toward the examined information sources, with the lowest proportion of participants perceiving almost all sources as reliable compared to other ethnic groups. Indians and Indigenous groups show similar levels of perceived reliability for investigated sources with a few exceptions. A notably high proportion of Indians (almost 80%) perceive NGOs as reliable compared to all other ethnic groups. Finally, slightly more than half of surveyed Indigenous groups perceived religious leaders as reliable, which is substantially higher compared to Indians and Chinese.

**Fig 2:**
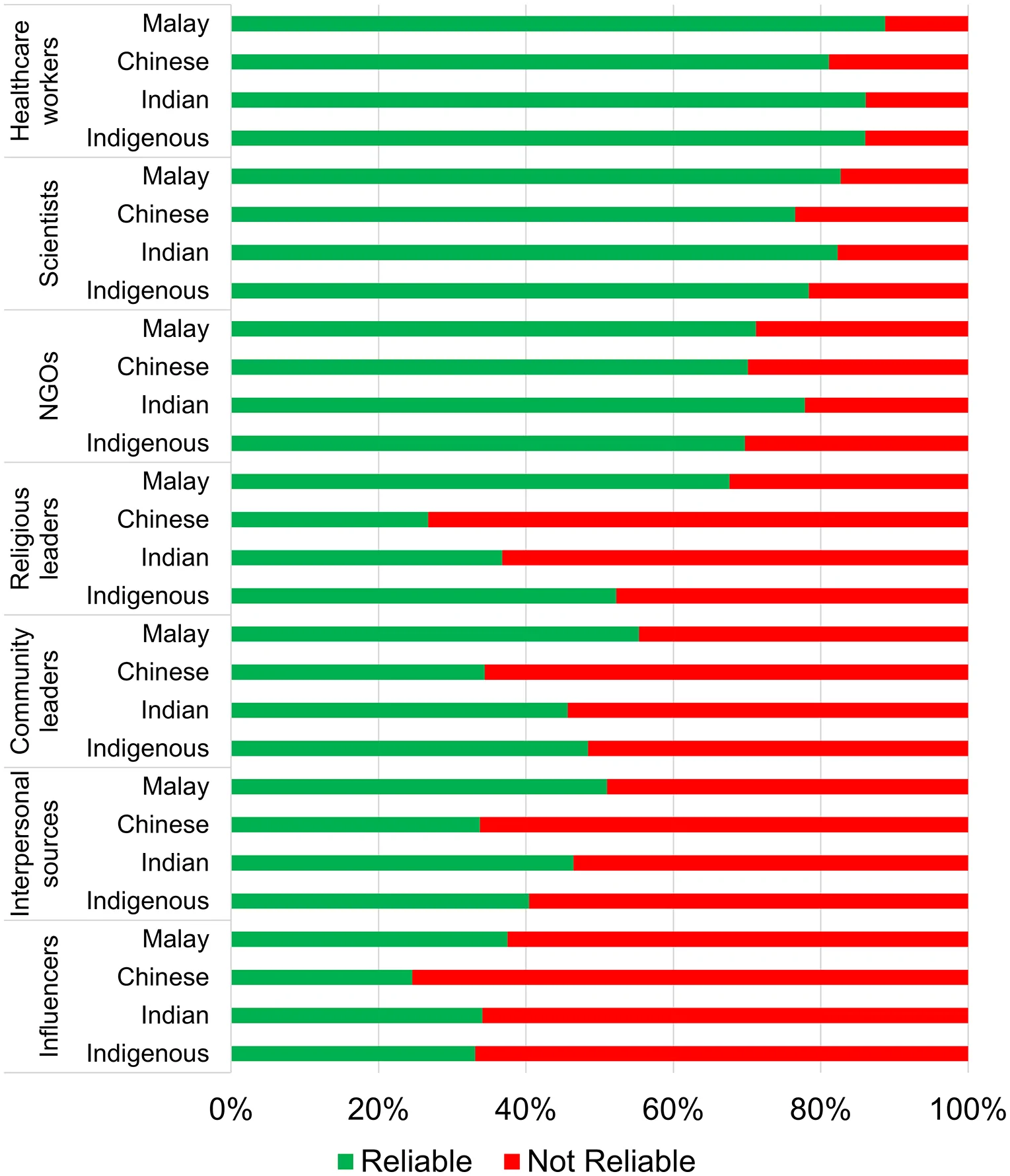
Proportion of participants’ perceived reliability of different information sources across ethnicities.

In the primary multivariable logistic regression model accounting for all studied sources while adjusting for general attitude toward vaccines (Table 2), four sources were significantly associated with the intention to vaccinate when perceived as reliable: healthcare workers, scientists, NGOs, and community leaders. Participants who perceived healthcare workers and NGOs as reliable sources of COVID-19 information had more than twofold higher odds of intending to receive the COVID-19 vaccine (Healthcare workers: OR = 2.07; 95% CI [1.60–2.68]; p < 0.001; NGOs: OR = 2.53; 95% CI [2.00–3.19]; p < 0.001). The odds of intending to receive the COVID-19 vaccine were approximately 70% higher among participants who perceived scientists as reliable sources (OR = 1.72; 95% CI [1.34–2.20]; p < 0.001) and approximately 50% higher among those who perceived community leaders as reliable sources (OR = 1.56; 95% CI [1.15–2.11]; p < 0.01). These findings remained fairly robust after controlling for additional sociodemographic variables, as shown in the second regression model in Table 2. As a sensitivity analysis, logistic regression analyses were repeated using the original 5-point Likert scale measures of perceived reliability for each source as predictors. The findings were largely consistent with those of the primary analyses, supporting the robustness of the results (Table D in S1 Appendix).

**Table 2:** Multivariable logistic regression modelling intention to receive COVID-19 vaccination based on perceived reliability of COVID-19 information sources.

	(1)^a^	(2)^b^
	Intention	Intention
Healthcare	2.074^***^	1.780^***^
workers	(0.272)	(0.250)
Scientists	1.719^***^	1.582^***^
	(0.217)	(0.207)
NGOs	2.528^***^	2.221^***^
	(0.300)	(0.274)
Religious	0.847	0.862
leaders	(0.114)	(0.123)
Community	1.557^**^	1.412^*^
leaders	(0.242)	(0.232)
Interpersonal	0.945	0.934
sources	(0.127)	(0.129)
Influencers	0.842	0.818
	(0.132)	(0.133)
Negative attitude	0.178^***^	0.215^***^
towards vaccines	(0.0216)	(0.0263)
Sex		1.002
		(0.109)
Age group		1.161^**^
		(0.0565)
Education		1.416^***^
level		(0.0999)
Income		1.281^**^
level		(0.103)
Trust in		2.438^***^
government		(0.310)
*N*	5784	5784

Exponentiated coefficients; Standard errors in parentheses.

* *p* < 0.05, ** *p* < 0.01, *** *p* < 0.001.

a. Multivariate logistic regression model accounting for all studied sources, adjusted for general attitude toward vaccines.

b. Multivariate logistic regression model accounting for all studied sources, adjusted for general attitude toward vaccines and other sociodemographic factors.

Fig 3 illustrates the adjusted predicted probabilities of each ethnic group perceiving information sources positively associated with vaccination intention as reliable, across education levels and age groups. The corresponding numerical estimates and 95% confidence intervals are presented in Tables E and F in S1 Appendix.

**Fig 3:**
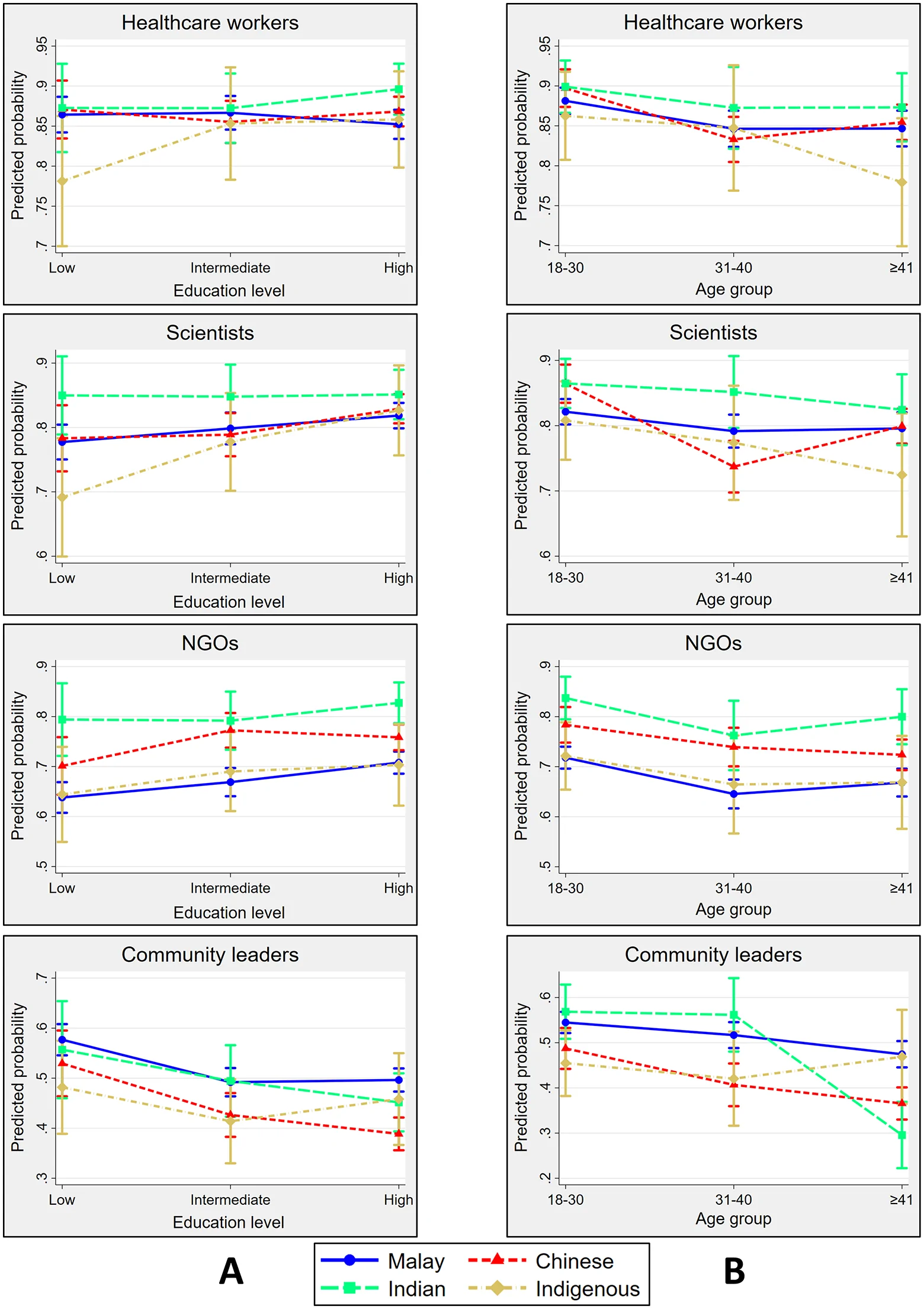
Predicted probability of perceiving information sources positively associated with vaccination intention as reliable across (A) different education levels and (B) different age groups, stratified by ethnic group (Malay, Chinese, Indian, and Indigenous). Points represent adjusted predicted probabilities derived from multivariable logistic regression models, and error bars indicate 95% confidence intervals.

Overall, the predicted probabilities were consistently above 85% for perceiving healthcare workers as reliable sources across all ethnicities regardless of education level, except for Indigenous groups. Those with a low education attainment had a significantly lower predicted probability of 78.13% (95% CI [70.01–86.25]), but this probability converged with other ethnicities from intermediate education levels onward. A similar trend in predicted probability was observed from an age group perspective, with predicted probabilities hovering between 83 – 90% for all ethnicities, regardless of age group except for Indigenous groups. Their predicted probability declined to 77.94% (95% CI [69.92–85.95]) among the oldest age group.

Indian respondents maintained a high and stable predicted probability of above 80% for perceiving Scientists as reliable sources, regardless of education level and age group. Across education levels, the Malays and Chinese shared an almost identical pattern of predicted probability—gently rising from approximately 78% at the lowest education level (Malays: Predicted probability = 77.74%; 95% CI [75.04–80.43]; Chinese: Predicted probability = 78.32%; 95% CI [73.19–83.46]) to 82% at the highest education level (Malays: Predicted probability = 81.84%; 95% CI [79.87–83.82]; Chinese: Predicted probability = 82.91%; 95% CI [80.64–85.17]). Indigenous groups with low education levels had much lower probabilities of perceiving Scientists as reliable (Predicted probability = 69.15%; 95% CI [59.96–78.34]) before converging with Malays and Chinese among respondents with intermediate levels of education.

Malays, Chinese, and Indigenous groups also shared similar predicted probabilities exhibiting a perceived reliability of scientists of above 80% in the youngest age category (Malays: Predicted probability = 82.13%; 95% CI [80.19–84.08]; Chinese: Predicted probability = 86.43%; 95% CI [83.50–89.35]; Indigenous groups: Predicted probability = 80.78%; 95% CI [74.77–86.79]). However, the predicted probability steeply declined to 73.71% (95% CI [69.76–77.67]), among Chinese in the 31– 40 age group, but this probability rebounded to 79.92%; 95% CI [77.25–82.59]) in the oldest age group. Malays in contrast maintained a predicted probability that hovered at approximately 80% across the older age categories (Malays: Predicted probability at age 31–40 = 79.16%; 95% CI [76.64–81.69]; Predicted probability at age ≥ 41 = 79.57%; 95% CI [77.12–82.02]). The predicted probability for Indigenous groups steadily declined to 77.37% (95% CI [68.62–86.13]) among the 31– 40 age group, and 72.46% (95% CI [63.03–81.88]) among the oldest age group.

Next, the predicted probability of perceiving NGOs as reliable sources, regardless of education level and age group, was highest among Indians, followed by Chinese, with both ethnicities averaging approximately 80% and 75%, respectively. Conversely, Malays and Indigenous groups shared similar patterns of having significantly lower predicted probabilities across all education levels and age groups, with point estimates of approximately 65% to 70%.

At the lowest education level and among the youngest age group, all ethnic groups had moderate predicted probabilities of perceiving information from community leaders as reliable, ranging from approximately 45% to 55%. As education and age increased, distinct patterns emerged across ethnic groups.

For Chinese respondents, the predicted probability of perceiving community leaders as reliable declined steadily with higher education and older age, dropping by more than 10 percentage points from the lowest to the highest level. Indian respondents showed a similar decrease with increasing education. However, their probability remained stable at approximately 56% across the two younger age groups before falling sharply to 29.57% (95% CI [22.23–36.90]) in the oldest group. Among Malays and Indigenous groups, the predicted probability decreased slightly at the intermediate education level by about 5 percentage points to 49.20% (95% CI [46.37–52.03]) and 41.40% (95% CI [33.00–49.80]) respectively, before stabilising between 45% and 50% at the high education level (Malays: Predicted probability = 49.64%; 95% CI [47.32–51.95]; Indigenous groups: Predicted probability = 45.83%; 95% CI [36.66–54.99]). Indigenous groups maintained relatively consistent probabilities across all age groups, averaging around 45%. Malays, on the other hand, experienced a slight probability decline to 51.69% (95% CI [48.83–54.55]) among the age 31 – 40 group, and 47.46% (95% CI [44.54–50.37]) among the oldest age group.

Diagnostic assessments of all multivariable logistic regression models indicated no evidence of problematic multicollinearity and adequate model fit, with results presented in Table G in S1 Appendix.

## Discussion

Our results suggest that all three formal information sources examined were widely perceived as reliable across all ethnic groups and were identified as information sources positively associated with vaccination intention. In contrast, among the informal sources, only religious and community leaders were viewed somewhat favourably, particularly by Malays compared to other ethnic groups. However, community leaders emerged as the only informal source identified as positively influencing vaccination intention. A more detailed examination within each ethnic group revealed additional nuances in how the reliability of these identified sources was perceived, varying according to education level and age group.

From the onset of the COVID-19 pandemic in March 2020, vast amounts of COVID-19–related health information were disseminated daily by a wide range of sources [22,34]. COVID-19 vaccines were also novel at the time of the study, and information regarding their development, safety, and benefits were disseminated through various sources prior to the vaccine rollout period [35,36]. Repeated exposure to COVID-19 information from a wide range of sources, coupled with perceptions of each source’s credibility, may have contributed to shaping public perceptions of which sources were perceived to provide reliable information worth acting upon. Consistent with Source Credibility Theory, these perceptions may help explain the observed associations between perceived reliability of information sources and COVID-19 vaccination intention [4].

We identified a robust positive association between perceiving healthcare workers, scientists, and NGOs as reliable sources of COVID-19 information and vaccination intentions, an association that has been reported across populations worldwide [37–40]. These findings further reinforce the critical role played by these professional and institutional groups in shaping public health responses.

The positive associations observed with healthcare workers likely stem from the trust conferred by society to a profession deemed as professionally trained, capable of providing credible evidence-based health information [41]. Perceiving scientists as reliable information sources on the other hand is associated with having trust in science or scientific sources, which is a key factor in conferring confidence to vaccinate [16,42]. Although the present study did not distinguish between types of NGOs, several mechanisms may explain why NGOs were perceived as reliable information sources and associated with vaccination intentions. International health and humanitarian organisations, exemplified by the World Health Organization (WHO) and the International Federation of Red Cross and Red Crescent Societies, may be perceived as credible due to their expertise and active role in disseminating evidence-based information to the community [43,44]. Meanwhile local NGOs may enhance accessibility by adapting health messages to local community contexts [45]. These mechanisms may help explain why NGOs were identified as an information source whose perceived reliability was positively associated with vaccination intention.

Perceptions of healthcare workers and scientists as highly reliable information sources were consistent among Malays, Chinese, and Indians, indicating that these professional groups command broad trust across Malaysia, a pattern that aligns with global survey findings [46]. The active and visible roles played by these formal information sources could have also generated a broad, cross-demographic “rally ‘round the flag” effect where institutional and authoritative figures receive heightened confidence in times of crisis, resulting in the wide reception for reliability among the surveyed population [47].

Although perceived reliability for healthcare workers and scientists remained generally high among Indigenous respondents, interaction analyses indicated that older age and low educational attainment were associated with modest reductions in the likelihood of perceiving these sources as reliable. These within-group differences may reflect variation in access to formal health communication, healthcare experiences, and opportunities to engage with professional health information among certain Indigenous subgroups, potentially shaping perceptions of these sources. Individuals living in rural or resource-limited settings may face greater barriers to healthcare access and engagement with formal institutions [48,49], while informal sources may often serve as primary channels of information [50,51]. However, these potential mechanisms were not directly examined in the present study and require further qualitative investigation.

Further examination of sociodemographic differences within Indigenous respondents revealed that education may represent an important factor underlying variations in perceiving healthcare workers and scientists as reliable. Higher education levels were associated with greater perceived reliability of these two sources. This observation could be linked to more educated individuals being more exposed to mainstream media, scientific discourse, and health information materials, thereby fostering greater familiarity and confidence in professional sources. This pattern aligns with evidence showing that education strengthens trust in medical and scientific authority by improving health literacy and the capacity to critically evaluate information [52–55]. In contrast, this association was not evident among other ethnicities, suggesting that trust in professional sources may already be established and less dependent on education. This difference could stem from higher baseline exposure to institutional health communication among these groups, potentially facilitated by greater urbanisation and access to healthcare infrastructure compared to Indigenous communities [56,57]. Further investigations are required to verify these interpretations.

Notably, a marked decline in the perceived reliability of both healthcare workers and scientists was observed among middle aged (age 31-40) Chinese. This pattern may indicate a stronger scepticism and critical evaluation that becomes more pronounced during this life stage within this group. Further research is warranted to explore the sociocultural, informational, and contextual factors underlying this distinct trend and to clarify how these dynamics differ from those observed in other ethnic groups.

The consistently higher perceived reliability of NGOs among Indians and Chinese, compared with Malays and Indigenous groups, may reflect broader structural determinants shaping public trust in information sources. Our survey also revealed ethnic disparities in perceptions of the government’s crisis management: smaller proportions of Chinese and Indians expressed trust compared with the other ethnic groups (see Fig A in S1 Appendix). These findings echo those of another Malaysian study, which reported that people who sourced information from the WHO were less likely to express confidence in the government’s management of the COVID-19 pandemic [34].

From an ethnic perspective, the Chinese and Indians constitute minority populations in Malaysia, whereas Malays and Indigenous groups are categorised as Bumiputera, the politically and socioeconomically dominant groups. Previous studies have shown that minority groups in Malaysia and other countries often report lower institutional trust, influenced by historical experiences, differences in policy orientation, and unequal levels of representation within public institutions [58–62]. These sociocultural dynamics may have contributed to the higher perceived reliability of NGOs among the Chinese and Indians. Previous research has suggested that lower institutional trust is associated with lesser reliance on government information sources [63], which may lead individuals to perceive non-governmental sources as more impartial and transparent [64]. However, this interpretation remains speculative, and further qualitative research is needed to explore the underlying reasons for these subgroup differences.

In contrast to formal sources, when examining informal sources, only community leaders reveal a significant association with positive COVID-19 vaccination intentions when perceived as reliable. Their influence on health behaviours may arise due to their role as intermediaries between formal institutions and the public. They facilitate top-down communication from authorities into culturally appropriate and relatable information that resonates with the local community, thus reducing information asymmetry and enhancing the relevance and acceptability of health messages within local contexts [65].

Respondents with low education levels exhibited a moderate probability of perceiving community leaders to be reliable. This finding reflects the capacity of community leaders to resonate with individuals having lower educational levels who are comfortable relying on community-based networks that are perceived as culturally aligned and familiar for information and guidance [66,67]. However, variations emerged across ethnic groups as education level and age increased.

Among the Chinese and Indians, the perceived reliability of community leaders declined steadily with education, reflecting a shift in information-seeking preferences as individuals with higher health literacy and critical appraisal skills are better able to comprehend and appreciate information from formal or expert-based sources [53,68]. Additionally, these ethnic groups displayed a similar trend as they aged. This pattern may again reflect the prevailing institutional distrust within these ethnic groups which is amplified among older individuals who have cumulatively greater exposure to conflicting or politicised information, which reduces trust towards local community leaders who are viewed as aligned with local institutional or political structures. These observations were in contrast to Malays and Indigenous groups. They displayed a decline in perceived reliability towards community leaders at intermediate education levels, which then stabilised at high education levels, while also portraying a relatively stable perceived reliability across different age groups as compared to the other two ethnicities. These trends may indicate two underlying factors. Firstly, it could be that these two ethnic groups have greater contact with community leaders who actively contribute in social and cultural affairs within their local community structure. Secondly, communal bonds arising from entrenched social systems which value interdependence and respect for communal authority may remain influential within these specific ethnic groups. Further studies are required to explore and verify these differences across the *Bumiputera* and minority groups.

Religious leaders were perceived as the most reliable source of COVID-19 information among all other informal sources, especially among the Malays. Malays, who are predominantly Muslim, tend to exhibit higher levels of religiosity compared to other ethnic groups [69], which may contribute to their stronger trust in religious authorities. This heightened religiosity is likely influenced by the prominent role of Islam in governance and public life, as well as an education system that promotes early and sustained engagement with religious teachings. However, despite being relatively well-received, religious leaders were not associated with COVID-19 vaccination intentions. Although highly trusted for spiritual and moral guidance, they may not be perceived as credible experts in scientific or medical matters, subsequently limiting their influence on health-related decisions [41]. Additionally, vaccination stances may vary across different religious leaders, with some endorsing vaccines while others remaining neutral or hesitant [70,71]. This inconsistency may have weakened any potential association between the perceived reliability of religious leaders and vaccination intentions. Policy makers should attempt to leverage the influence of religious leaders by aligning them towards a common public health goal through active engagement and dialogue. Such alignment could harness their potential to positively influence health promotion efforts among communities where religion strongly shapes health beliefs and behaviours.

An additional consideration when interpreting the perceived reliability of informal information sources is that our study presented information sources as broad categories without specific information about the educational, professional, or health-related background of the assessed messengers. For example, religious or community leaders with health-related training or experience could potentially influence how their information is perceived. Examining whether providing such information about messenger characteristics influences perceived reliability was outside the scope of the present study but represents an interesting area for future research.

Perceived reliability was assessed using a single-item measure, which captured the overall evaluation of the reliability of COVID-19 information from each source. While single-item measures have been commonly used to assess overall perceptions of information sources [18,72–74], they do not distinguish between the underlying dimensions that contribute to these evaluations, such as perceived expertise, trustworthiness, or other attributes related to source credibility. Consequently, the present study was unable to determine which specific aspects of source credibility most strongly influenced perceptions of reliability. Additionally, although our logistic regressions yielded conveniently interpretable results that were proven robust via sensitivity analyses, dichotomising the 5-point Likert-scale measure of perceived reliability may have resulted in some loss of information and limited our ability to examine how sociodemographic factors were associated with varying levels of perceived reliability. Future research could employ multidimensional measures that retain the full range of response categories to examine these constructs separately and provide greater insight into the mechanisms underlying perceptions of information reliability and their associations with health behaviours.

Future research should also aim to explore deeper insights into the reasons and motivations behind the perceived reliability of different information sources through qualitative approaches. Such insights would provide clarity into the underlying mechanisms driving the trends observed in our study. Additionally, future studies could examine the specific sources that each ethnic group actively uses to seek health information and investigate their association with adopting broader health behaviours that extend beyond vaccination. Understanding the role of source selection and its impact on health-related actions will provide valuable insights for targeted health communication strategies.

This study has several limitations. First, participants were recruited through an online research panel, which may have introduced selection bias by preferentially including individuals with internet access, higher digital literacy, and a greater willingness to participate in online surveys. Consequently, the sample was skewed towards younger and more highly educated individuals, and Indigenous populations were underrepresented relative to the Malaysian population. These characteristics may reflect the digital divide in access to and participation in online surveys, potentially limiting the inclusion of socioeconomically disadvantaged and geographically remote populations. Hence, findings from this present study may not be fully generalisable to the Malaysian population and should be applied with caution, especially to rural, digitally underserved, and less educated populations. Nevertheless, the findings may remain relevant to individuals within these populations who actively engage with digital platforms and are exposed to similar sources of health information, although the extent to which this applies across different age groups, geographic settings, and levels of digital access remains uncertain.

The second limitation concerns the assumption that participants had prior exposure to COVID-19–related information from all sources examined due to health information being widespread during the pandemic. However, it remains uncertain whether responses reflected actual exposure to information from these sources or whether participants relied on inherent heuristics when responding. Third, the study relied on self-reported measures of perceived reliability and vaccination intention, which may be subject to measurement biases. Fourth, although analyses adjusted for several sociodemographic factors, residual confounding from unmeasured factors cannot be excluded. Additionally, response rate and information on excluded or incomplete responses were unavailable from the panel provider, limiting assessment of potential non-response bias. Lastly, given the cross-sectional design, temporal relationships cannot be established and reverse causality cannot be ruled out, as individuals who were already more inclined to receive the COVID-19 vaccine may have been more likely to perceive information from certain sources as reliable.

## Conclusion

Our study documents evidence of the diversity in the perceptions of reliable health information sources in a multi-ethnic society such as Malaysia. These findings suggest that, in the event of a health emergency, it is essential to understand the varying levels of credibility placed in various messengers among different communities.

Healthcare workers and scientists demonstrated consistently high perceived reliability across most groups, suggesting that they should remain central figures in national health communication strategies. To further strengthen their impact, focused initiatives are needed to improve health literacy and awareness among specific ethnic groups, particularly Indigenous groups, who may experience lower exposure to or trust in these formal information sources.

At the same time, NGOs and community leaders show potential to connect with particular segments of the population through structural trust, cultural affinity, and community engagement. These actors may play complementary roles in reinforcing public health communication and bridging informational disparities across groups.

Taken together, these findings support an integrated, multi-messenger approach that engages both formal and certain informal information sources, recognising that the perceived reliability of these sources may vary across population groups. Such an approach may help health communication stakeholders select and engage appropriate sources when developing communication strategies to maximise outreach and support effective responses during public health crises.

## Supporting information

S1 Appendix Table A: Association between study arms and perceived reliability of COVID-19 information sources. **Table B:** Proportion of participants’ perceived reliability of different information sources. **Table C:** Proportion of participants’ perceived reliability of different information sources across ethnicities. **Table D:** Multivariable logistic regression model of intention to receive COVID-19 vaccination based on the undichotomised 5-point Likert scale measure of perceived reliability of COVID-19 information sources. **Table E:** Predicted probabilities (%) of perceiving information sources positively associated with vaccination intention as reliable, by ethnicity across education levels. **Table F:** Predicted probabilities (%) of perceiving information sources positively associated with vaccination intention as reliable, by ethnicity across age groups. **Table G:** Multicollinearity and Goodness-of-fit assessment for the multivariable logistic regression models predicting COVID-19 vaccination intention and perceived reliability of specific COVID-19 information sources. **Fig A:** Proportion of respondents across ethnicities reporting trust in the government’s management of COVID-19.
(DOCX)
