# Supplementary material for: Does the messenger make the difference? Perceived reliability of health information sources during the COVID-19 pandemic in multi-ethnic Malaysia
Source: PLOS Glob Public Health. 2026 Oct 1;6(10):e0007397. doi: 10.1371/journal.pgph.0007397 (PMC13630309; doi:10.1371/journal.pgph.0007397)
Supplement: Supplementary file 1 [file pgph.0007397.s001.docx]

**S1 Appendix**

**Table A:** Association between study arms and perceived reliability of COVID-19 information sources.

| **Source of health information** | **χ² statistic** | **p-value** |
| --- | --- | --- |
| Healthcare workers | 9.1733 | 0.760 |
| Scientists | 12.8019 | 0.463 |
| NGOs | 12.5137 | 0.486 |
| Religious leaders | 8.6426 | 0.799 |
| Community leaders | 5.4120 | 0.965 |
| Interpersonal sources | 11.8771 | 0.538 |
| Influencers | 20.2325 | 0.090 |

Note: Degrees of freedom for all Pearson's chi-square tests were 13, based on 14 study arms and two categories of perceived reliability (i.e., reliable and not reliable).

**Table B:** Proportion of participants' perceived reliability of different information sources.

|  | **Perceived reliability of COVID-19 information** | | | | |
| --- | --- | --- | --- | --- | --- |
| **Source of health information** | **Strongly Not Reliable  (%)** | **Not Reliable (%)** | **Not Sure (%)** | **Reliable (%)** | **Strongly Reliable (%)** |
| Healthcare workers | 0.5 | 1.9 | 11.3 | 54.1 | 32.2 |
| Scientists | 0.7 | 2.3 | 16.3 | 54.9 | 25.8 |
| Non-governmental organisations | 1.8 | 3.8 | 22.9 | 49.7 | 21.8 |
| Religious leaders | 5.3 | 10.4 | 31.4 | 37.7 | 15.2 |
| Community leaders | 3.2 | 11.1 | 37.3 | 37.1 | 11.3 |
| Interpersonal sources | 1.6 | 10.8 | 42.2 | 35.7 | 9.6 |
| Influencers | 5.8 | 17.3 | 43.4 | 25.3 | 8.2 |

**Table C:** Proportion of participants' perceived reliability of different information sources across ethnicities.

|  |  | **Perceived reliability of COVID-19 information** | | | | |
| --- | --- | --- | --- | --- | --- | --- |
| **Source** | **Ethnicity** | **Strongly Not Reliable**  **(%)** | **Not Reliable**  **(%)** | **Not Sure**  **(%)** | **Reliable**  **(%)** | **Strongly Reliable**  **(%)** |
| Healthcare workers | Malay | 0.44 | 1.47 | 9.36 | 52.31 | 36.42 |
|  | Chinese | 0.44 | 2.66 | 15.77 | 58.64 | 22.48 |
|  | Indian | 1.35 | 2.12 | 10.40 | 55.11 | 31.02 |
|  | Indigenous | 0.35 | 1.74 | 11.85 | 48.78 | 37.28 |
| Scientists | Malay | 0.68 | 1.77 | 14.83 | 54.46 | 28.27 |
|  | Chinese | 0.57 | 2.91 | 20.01 | 57.31 | 19.19 |
|  | Indian | 1.35 | 2.89 | 13.49 | 53.18 | 29.09 |
|  | Indigenous | 0.00 | 3.48 | 18.12 | 50.52 | 27.87 |
| NGOs | Malay | 2.03 | 3.77 | 22.98 | 48.13 | 23.10 |
|  | Chinese | 1.20 | 3.80 | 24.89 | 53.39 | 16.72 |
|  | Indian | 2.12 | 4.62 | 15.41 | 49.52 | 28.32 |
|  | Indigenous | 2.09 | 3.14 | 25.09 | 47.74 | 21.95 |
| Religious leaders | Malay | 2.35 | 4.68 | 25.36 | 46.31 | 21.30 |
|  | Chinese | 9.88 | 20.58 | 42.81 | 22.61 | 4.12 |
|  | Indian | 10.79 | 18.69 | 33.72 | 27.94 | 8.86 |
|  | Indigenous | 4.18 | 6.62 | 36.93 | 37.28 | 14.98 |
| Community leaders | Malay | 2.74 | 8.03 | 33.89 | 40.89 | 14.45 |
|  | Chinese | 3.74 | 16.34 | 45.54 | 29.96 | 4.43 |
|  | Indian | 5.01 | 15.41 | 33.91 | 34.10 | 11.56 |
|  | Indigenous | 2.79 | 10.45 | 38.33 | 37.28 | 11.15 |
| Interpersonal sources | Malay | 1.29 | 8.24 | 39.45 | 39.63 | 11.39 |
|  | Chinese | 1.96 | 14.63 | 49.65 | 28.31 | 5.45 |
|  | Indian | 3.08 | 14.64 | 35.84 | 34.30 | 12.14 |
|  | Indigenous | 1.39 | 13.94 | 44.25 | 32.75 | 7.67 |
| Influencers | Malay | 5.41 | 14.27 | 42.81 | 27.24 | 10.27 |
|  | Chinese | 6.40 | 23.24 | 45.79 | 20.96 | 3.61 |
|  | Indian | 6.55 | 20.62 | 38.73 | 25.63 | 8.48 |
|  | Indigenous | 5.92 | 14.63 | 46.34 | 25.44 | 7.67 |

**Table D:** Multivariable logistic regression model of intention to receive COVID-19 vaccination based on the undichotomised 5-point Likert scale measure of perceived reliability of COVID-19 information sources.

|  | (1)^a^ | (2)^b^ |
| --- | --- | --- |
|  | Intention | Intention |
|  |  |  |
| Healthcare | 1.524^***^ | 1.413^***^ |
| workers | (0.133) | (0.126) |
|  |  |  |
| Scientists | 1.305^**^ | 1.282^**^ |
|  | (0.111) | (0.113) |
|  |  |  |
| NGOs | 1.708^***^ | 1.591^***^ |
|  | (0.117) | (0.112) |
|  |  |  |
| Religious | 0.975 | 1.018 |
| leaders | (0.0697) | (0.0772) |
|  |  |  |
| Community | 1.297^**^ | 1.255^*^ |
| leaders | (0.120) | (0.120) |
|  |  |  |
| Interpersonal | 0.933 | 0.922 |
| sources | (0.0806) | (0.0810) |
|  |  |  |
| Influencers | 0.938 | 0.941 |
|  | (0.0776) | (0.0803) |
|  |  |  |
| Negative attitude | 0.187^***^ | 0.228^***^ |
| towards vaccines | (0.0227) | (0.0280) |
|  |  |  |
| Sex |  | 0.977 |
|  |  | (0.107) |
|  |  |  |
| Age group |  | 1.203^***^ |
|  |  | (0.0589) |
|  |  |  |
| Education |  | 1.471^***^ |
| level |  | (0.106) |
|  |  |  |
| Income |  | 1.281^**^ |
| level |  | (0.105) |
|  |  |  |
| Trust in |  | 2.145^***^ |
| government |  | (0.266) |
| *N* | 5784 | 5784 |

Exponentiated coefficients; Standard errors in parentheses

^*^ *p* < 0.05, ^**^ *p* < 0.01, ^***^ *p* < 0.001

a. Primary multivariate logistic regression model accounting for all studied sources, adjusted for general attitude toward vaccines.
b. Multivariate logistic regression model accounting for all studied sources, adjusted for general attitude toward vaccines and other sociodemographic factors.

**Table E:** Predicted probabilities (%) of perceiving information sources positively associated with vaccination intention as reliable, by ethnicity across education levels.

|  | **Healthcare workers** |  | **Scientists** |  | **NGOs** |  | **Community leaders** |  |
| --- | --- | --- | --- | --- | --- | --- | --- | --- |
|  |  |  |  |  |  |  |  |  |
|  | Predicted probability  (%) | 95% CI | Predicted probability  (%) | 95% CI | Predicted probability  (%) | 95% CI | Predicted probability  (%) | 95% CI |
| Malays, Low | 86.44 | [84.21,88.66] | 77.74 | [75.04,80.43] | 63.81 | [60.74,66.88] | 57.69 | [54.57,60.80] |
| Malays, Intermediate | 86.66 | [84.56,88.77] | 79.86 | [77.40,82.32] | 66.89 | [64.07,69.71] | 49.20 | [46.37,52.03] |
| Malays, High | 85.22 | [83.40,87.04] | 81.84 | [79.87,83.82] | 70.79 | [68.57,73.00] | 49.64 | [47.32,51.95] |
| Chinese, Low | 87.07 | [83.46,90.69] | 78.32 | [73.19,83.46] | 70.13 | [64.37,75.88] | 52.95 | [46.37,59.52] |
| Chinese, Intermediate | 85.52 | [82.89,88.16] | 78.89 | [75.53,82.24] | 77.25 | [73.78,80.71] | 42.65 | [38.28,47.03] |
| Chinese, High | 86.83 | [84.98,88.68] | 82.91 | [80.64,85.17] | 75.85 | [73.26,78.44] | 38.86 | [35.59,42.13] |
| Indians, Low | 87.27 | [81.75,92.79] | 84.98 | [78.91,91.05] | 79.40 | [72.13,86.67] | 55.69 | [46.00,65.39] |
| Indian, Intermediate | 87.24 | [82.91,91.57] | 84.79 | [79.82,89.77] | 79.18 | [73.37,84.99] | 49.45 | [42.31,56.59] |
| Indians, High | 89.62 | [86.43,92.80] | 85.12 | [81.28,88.96] | 82.74 | [78.65,86.83] | 45.17 | [39.37,50.97] |
| Indigeneous, Low | 78.13 | [70.01,86.25] | 69.15 | [59.96,78.34] | 64.43 | [54.92,73.93] | 48.18 | [38.88,57.48] |
| Indigeneous, Intermediate | 85.32 | [78.31,92.34] | 77.76 | [70.16,85.35] | 69.02 | [61.09,76.94] | 41.40 | [33.00,49.80] |
| Indigeneous, High | 85.83 | [79.81,91.84] | 82.66 | [75.67,89.65] | 70.34 | [62.18,78.50] | 45.83 | [36.66,54.99] |
| Observations | 5784 |  | 5784 |  | 5784 |  | 5784 |  |

**Table F:** Predicted probabilities (%) of perceiving information sources positively associated with vaccination intention as reliable, by ethnicity across age groups.

|  | **Healthcare workers** |  | **Scientists** |  | **NGOs** |  | **Community leaders** | |  |
| --- | --- | --- | --- | --- | --- | --- | --- | --- | --- |
|  |  |  |  |  |  |  |  |  | |
|  | Predicted probability  (%) | 95% CI | Predicted probability  (%) | 95% CI | Predicted probability  (%) | 95% CI | Predicted probability  (%) | 95% CI | |
| Malay, 18-30 | 88.14 | [86.49,89.78] | 82.13 | [80.19,84.08] | 71.80 | [69.61,73.99] | 54.48 | [52.14,56.83] | |
| Malay, 31-40 | 84.63 | [82.37,86.90] | 79.16 | [76.64,81.69] | 64.55 | [61.67,67.43] | 51.69 | [48.83,54.55] | |
| Malay, ≥ 41 | 84.67 | [82.43,86.91] | 79.57 | [77.12,82.02] | 66.82 | [64.05,69.60] | 47.46 | [44.54,50.37] | |
| Chinese, 18-30 | 89.72 | [87.37,92.08] | 86.43 | [83.50,89.35] | 78.37 | [74.81,81.93] | 48.74 | [44.20,53.27] | |
| Chinese, 31-40 | 83.30 | [80.48,86.13] | 73.71 | [69.76,77.67] | 73.92 | [70.07,77.77] | 40.68 | [35.96,45.41] | |
| Chinese, ≥ 41 | 85.45 | [83.23,87.66] | 79.92 | [77.25,82.59] | 72.37 | [69.31,75.43] | 36.56 | [33.00,40.11] | |
| Indians, 18-30 | 89.91 | [86.63,93.19] | 86.47 | [82.70,90.24] | 83.72 | [79.47,87.98] | 56.86 | [50.86,62.87] | |
| Indians, 31-40 | 87.26 | [82.14,92.39] | 85.16 | [79.65,90.67] | 76.24 | [69.31,83.18] | 56.17 | [48.04,64.31] | |
| Indians, ≥ 41 | 87.32 | [83.05,91.60] | 82.45 | [77.03,87.87] | 79.98 | [74.49,85.48] | 29.57 | [22.23,36.90] | |
| Indigeneous,  18-30 | 86.26 | [80.75,91.77] | 80.78 | [74.77,86.79] | 72.15 | [65.42,78.88] | 45.49 | [38.21,52.77] | |
| Indigeneous,  31-40 | 84.74 | [76.89,92.59] | 77.37 | [68.62,86.13] | 66.44 | [56.65,76.24] | 42.04 | [31.63,52.46] | |
| Indigeneous, ≥ 41 | 77.94 | [69.92,85.95] | 72.46 | [63.03,81.88] | 66.87 | [57.58,76.16] | 46.89 | [36.49,57.28] | |
| Observations | 5784 |  | 5784 |  | 5784 |  | 5784 |  | |

**Table G:** Multicollinearity and Goodness-of-fit assessment for the multivariable logistic regression models predicting COVID-19 vaccination intention and perceived reliability of specific COVID-19 information sources.

| **Model outcome** | **Observations** | **VIF (mean; range)** | **Hosmer–Lemeshow χ² (df)** | **Hosmer–Lemeshow**  **p-value** |
| --- | --- | --- | --- | --- |
| Intention to receive COVID-19 vaccination (Primary model) | 5,784 | 1.58 (1.10 – 2.11) | 7.38 (7) | 0.3904 |
| Intention to receive COVID-19 vaccination (Second model) | 5,784 | 1.43 (1.04 – 2.15) | 7.99 (8) | 0.4347 |
| Perceiving Healthcare workers as reliable (Interaction between ethnicity and education level) | 5,784 | 3.11 (1.04 – 6.99) ^a^ | 8.15 (8) | 0.4188 |
| Perceiving Healthcare workers as reliable (Interaction between ethnicity and age group) | 5,784 | 1.84 (1.04 – 3.44) | 7.18 (8) | 0.5173 |
| Perceiving Scientists as reliable  (Interaction between ethnicity and education level) | 5,784 | 3.11 (1.04 – 6.99) ^a^ | 6.11 (8) | 0.6347 |
| Perceiving Scientists as reliable  (Interaction between ethnicity and age group) | 5,784 | 1.84 (1.04 – 3.44) | 13.80 (8) | 0.0870 |
| Perceiving NGOs as reliable  (Interaction between ethnicity and education level) | 5,784 | 3.11 (1.04 – 6.99) ^a^ | 25.59 (8) | 0.001^b^ |
| Perceiving NGOs as reliable  (Interaction between ethnicity and age group) | 5,784 | 1.84 (1.04 – 3.44) | 24.07 (8) | 0.002 ^b^ |
| Perceiving Community leaders as reliable  (Interaction between ethnicity and education level) | 5,784 | 3.11 (1.04 – 6.99) ^a^ | 5.42 (8) | 0.7119 |
| Perceiving Community leaders as reliable  (Interaction between ethnicity and age group) | 5,784 | 1.84 (1.04 – 3.44) | 5.26 (8) | 0.7296 |

a. Although some categorical ethnicity terms demonstrated VIF values above, these elevations were observed in the context of ethnicity-by-education interaction terms and were expected due to the inclusion of correlated indicator variables. The overall mean VIF remained low and no substantial evidence of problematic multicollinearity was identified.
b. Although the Hosmer–Lemeshow test was statistically significant for the NGO model, the large sample size may result in sensitivity to minor deviations from calibration. The model demonstrated acceptable discrimination (AUC = 0.71), and interpretation focused on predicted probabilities derived from the regression model.

**Fig A.** Proportion of respondents across ethnicities reporting trust in the government's management of COVID-19.
